# Associations between Specialist Tactical Response Police Unit Selection Success and Urban Rush, along with 2.4 km and 10 km Loaded Carriage Events

**DOI:** 10.3390/ijerph16193558

**Published:** 2019-09-23

**Authors:** Rhiannon Thomas, Ben Schram, Shane Irving, Jeremy Robinson, Robin Orr

**Affiliations:** 1 Physiotherapy Department, Faculty of Health Sciences and Medicine, Bond University, Gold Coast 4226, Australia; rhiannon.thomas@student.bond.edu.au (R.T.); bschram@bond.edu.au (B.S.); 2 Tactical Research Unit, Bond University, Gold Coast 4226, Australia; sirving@bond.edu.au; 3 Australian Federal Police, Canberra 2601, Australia; Jeremy.Robinson@afp.gov.au

**Keywords:** police, SWAT, tactical, fitness, selection

## Abstract

Officers serving in specialist tactical response police teams are highly trained personnel who are required to carry heavy loads and perform explosive tasks. The aim of this study was to determine whether performance on a loaded explosive occupational task (urban rush) or distance-based load carriage tasks (2.4 km or 10 km) were indicative of officer success on a specialist selection course (SSC). Eighteen male police officers (mean age = 32.11 ± 5.04 years) participated in the SSC over five consecutive days. Data were categorized into Group 1 (successful applicants, n = 11) and Group 2 (unsuccessful applicants, n = 7). Independent sample *t*-tests were performed to determine differences between groups, along with point-biserial correlations to investigate associations between anthropometric and event performance data and course completion success. Alpha levels were set at *p* = 0.05 a priori. Height (*p* = 0.025), body weight (*p* = 0.007), and 2.4 km loaded performance (*p* = 0.013) were significantly different between groups, where being shorter (r_pb_(16) = −0.526, *p* < 0.05), lighter (r_pb_(16) = −0.615, *p* < 0.01), and faster (r_pb_(16) = −0.572, *p* < 0.05) were associated with course success. While a loaded 2.4 km event is associated with success, a ceiling effect for an explosive anaerobic task and a longer 10 km task may exist, whereby increases in performance are not associated with selection success.

## 1. Introduction

Tactical populations, inclusive of law enforcement, firefighters, and military personnel, are required to perform a variety of occupationally specific and physically demanding tasks under challenging and variable conditions [1,2,3]. To complete the intense training and subsequently be successful in their fields of work, tactical personnel are required to possess a requisite level of physical fitness [2,4,5,6].

Police officers who serve as members of specialist tactical response police units, such as those in a special weapons and tactics (SWAT) unit, are highly trained personnel who are at the forefront of maintaining national security [7,8,9]. At any given time during their work day, personnel serving on specialist tactical response teams may be required to complete hostage rescue missions, fugitive apprehensions, and respond to national acts of terrorism [7,10]. For these officers to complete high-risk operations, they require additional training to acquire the higher level of physical fitness and technical skills needed, which are above those of other general tactical officers [7,11]. Identifying personnel capable of being trained for, and being able to serve on, specialist tactical response teams is usually undertaken through specialist selection processes [12,13].

The intensive training required to be a specialist tactical response police officer can lead to an increased risk of injury as heavier than typical loads are carried on the body [14,15] and new physical tasks of a higher complexity [13] are introduced at greater absolute workloads to ready the officers for active duty [16]. The additional physical demands placed on these personnel can result in overtraining and jeopardize their ability to successfully complete the training process, or in the long term can impact on their ability to perform optimally during operational tasks [13,17].

The ability of specialist tactical response personnel to carry loads is of importance [18,19], given that they are required to carry heavy loads comprising specialized operational equipment (specialist weapons, ballistic shields, armour, etc.) whilst performing their tasks [20]. General police officers can carry average daily duty loads of up to 10 kg, with body armour adding additional weight [21,22]. In specialist police units, personnel can be required to carry loads ranging from 23 kg up to 40 kg while completing operations [23,24]. Although essential, the carriage of these loads can lead to an increased risk of injury, as well as a negative impact on task performance [25]. For example, heavy load carriage (up to 22.8 kg) [26] has been found to reduce the ability of specialist tactical response police personnel to traverse terrain, and to rescue and drag civilians to safety [13,27]. Additionally, load carriage may impact on the marksmanship skills of tactical personnel [13,23,26,28,29]. One means of mitigating these performance risks is to train with these occupational loads, with research showing a minimal impact of load carriage on SWAT officers who constantly trained while wearing their loads [26]. These findings by Carlton and colleagues [26] highlight how the requirement to carry loads for specialist tactical response police extends beyond performing occupational tasks to form part of their ongoing specialist training [30].

Given the importance of load carriage tasks for specialist personnel, these tasks often form part of most specialist selection processes [12,13]. Although performance in long distance load carriage tasks (ranging from 3.2 km to 20 km in distance) [12,13,31] are commonly used as a selection criterion, the use of short explosive load carriage events is uncommon, and hence the literature is limited in this area [32]. A study by Treloar et al. [19] in a military population found the addition of 21.6 kg in load to infantry soldiers increased sprint times by 31.5% for every sprint bout completed when compared to their unloaded times. As the soldiers are required to perform multiple short sprints in an operational environment, this cumulative decrement in performance equates to a significant reduction in movement speed while engaging the enemy and could substantially affect a soldier’s survivability [19]. These short, explosive tasks may likewise be required by specialist tactical response police during armed offender incidents or when seeking cover after exiting a vehicle [33]. Therefore, the aim of this study was to determine whether performance on a loaded explosive occupational task (the urban rush (UR)), as well as longer 2.4 km and 10 km load carriage tasks, is indicative of candidate success on a police specialist selection course (SSC).

## 2. Materials and Methods

### 2.1. Officers

Retrospective data were provided for 18 male police officers (mean age = 32.11 ± 5.04 years, mean height = 183.72 ± 7.9 cm, mean weight = 89.44 ± 8.56 kg, mean body mass index (BMI) = 26.39 ± 1.58 kg/m^2^, and mean loaded BMI (LBMI) = 35.60 ± 1.61 kg/m^2^) who attempted a police SSC. The Bond University Human Research Ethics Committee approved this study (# 15412).

### 2.2. Protocols

This study was a retrospective cohort study, with data provided by the specialist police agency where the SSC took place. The protocols for the study were those in use by the agency as part of their workplace processes, and are described below.

### 2.3. Age and Height

The age (years) and height (centimeters) of the officers were self-reported. The use of self-reported height data has been successfully used previously in this population [5] and is considered a reliable [34] and valid measure [5]. Preceding the loaded carriage events, the officers’ body weights were obtained (using Tanita BC82 Fitplus scales) whilst in shorts and shirts, without footwear.

### 2.4. BMI and Loaded BMI

BMI was determined through use of the formula $BMI=\frac{weight}{height^{2}}$ [35]. In addition to BMI, a novel measure, loaded BMI (LBMI), was created. LBMI modified the mass measure to include the external load carried by the officer (*L* $BMI=\frac{loaded\;weight}{height^{2}}$). This measure was a trial modification to see if the external load, when considered with the individual’s body weight, could be a potentially viable measure for these personnel in the future.

### 2.5. Urban Rush

The test was conducted on a flat, dry, non-slip surface with a series of parallel lines and cones marking the start, 10 m, and 20 m lines respectively. The officers started in a standing position behind the starting line dressed in their operational uniform for a “black” or urban role (mean load weight 28.43 ± 0.54 kg), consisting of a 17.5 kg plate carrier, 3.6 kg primary weapon, and all accessories. On the command of the assessor, the officers ran to the first 10 m parallel line, dropped into a prone position, and crawled from the 10 m to the 20 m line. On reaching the 20 m line, the officer stood, slung their weapon and raised an 80 kg mannequin off the ground into a drag position. The mannequin was then dragged backwards to the previous 10 m line. Upon arrival at the 10 m line, the officer lowered the mannequin and adopted a kneeling unsupported position, raised the muzzle of their weapon and aligned the laser targeting system of their weapon with a humanoid target. Once completed, the weapon was again slung, and the mannequin was picked up again and dragged to the starting line. Once again, the officer lowered the mannequin and adopted the kneeling firing position and aligned the laser with the target. Officers were then required to sprint back to the first 10 m line and again adopt the unsupported kneel position and aim at the target. This task was then repeated to the 20 m line, where the officers completed a 180-degree pivot, lowered to the unsupported kneeling position and aligned their targeting laser with a second target behind the starting line. Standing, the officer ran to the 10 m line and performed the same kneeling and targeting action before running another 10 m to the starting line where the mannequin was now situated. The officer then stood, picked up the mannequin and dragged it the full 20 m where they again dropped to one knee and aligned their sights with the target. The officers then sprinted a final 20 m to the starting line, where the assessor ceased timing the test once they had crossed the starting line. Each time the officer aligned the laser sighting system with a target, they were not allowed to continue the course unless a verbal acknowledgement was given by the assessor that the weapon system was correctly aligned with the target. The event was to be completed as fast as possible, and was timed, in seconds, by a strength and conditioning coach on a hand-held stopwatch (Hart Sport).

### 2.6. 2.4 km and 10 km Loaded Marches

The officers were issued with standard operational boots and camouflage uniforms. Officers were required to carry a 17.5 kg plate carrier (2.4 km) or backpack loaded with 25 kg (10 km) (measured using a Wedderburn (Canberra, Australia) Ds-530 Digital Industrial Scale) and carry an unloaded weapon weighing 3.6 kg (both events). The 2.4 km loaded movement and 10 km loaded march were completed on a flat surface and consisted of a combination of hard soil and bitumen. The route of the assessment was not disclosed due to security requirements, given that the location was an operational training area. However, the route was marked using a handheld Garmin Oregon 600t GPS system (Garmin, Kansas City, USA). The duration taken to complete the loaded events were recorded by a strength and conditioning coach using a handheld timer (Junsd 50, Hart Sport (Aspley, Australia)). The officers had been given clearance to jog during the assessment, as it was reflective of tasks they may face in operations in the field.

### 2.7. The Selection Process and Determining Success or Failure

The selection process was conducted over a 5-day period after all physical performance testing. Over this timeframe, the officers were required to complete 97 events, all of which had different competency and performance measures and required varying intensities and modalities of fitness. All events in the selection process were designed to determine whether the officer was deemed suitable to commence training as a specialist tactical response police officer. An officer could be medically withdrawn, could self-withdraw, or be removed from the course by directing staff at any stage during the course if deemed to not be performing suitably prior to the completion of the selection course.

The assessment items ranged from physical exercises to various operational scenarios, where the officers were assessed on their critical decision-making skills under stressful conditions, with the accumulative effects of sleep deprivation, load carriage, and cognitive and physical fatigue due to incessant events, along with continuous performance anxiety caused by the environment. The officers were also required to complete a series of load carriage events with variable terrain or topography in urban and rural settings, in addition to high intensity anaerobic tasks with minimal recovery provided. Lastly, obstacle courses, water proofing events, and high angle roping activities were also completed with no additional weight worn.

### 2.8. Statistical Analysis

The non-identifiable data were provided on a Microsoft Excel spreadsheet and was subsequently imported into the Statistical Package for the Social Sciences (IBM SPSS, Armonk, USA, version 24) for analysis. Independent sample *t*-tests were conducted for all anthropometric and loaded event data to determine whether any differences existed between those personnel who were unsuccessful or successful in selection after normality, determined via a Shapiro–Wilk test and with equality of variances ensured with the Levene’s test. Additionally, point-biserial correlations were conducted to determine associations between anthropometric and performance variables and course success. Alpha levels were set at *p* = 0.05 *a priori*.

## 3. Results

Of the 18 officers attending the SSC, seven officers (mean age = 34.43 ± 4.54 years, mean height = 187.43 ± 4.58 cm, mean body weight = 95.86 ± 7.45 kg, mean BMI 27.27 ± 1.46 kg/m^2^, and mean LBMI= 34.02 ± 1.38 kg/m^2^) did not complete the selection process due to injury or self-withdrawal. This left eleven officers (mean age = 30.64 ± 4.97 years, mean height = 181.36 ± 5.35 cm, mean body weight = 85.36 ± 6.65 kg, mean BMI = 25.94 ± 1.49 kg/m^2^, and mean LBMI= 33.33 ± 1.75 kg/m^2^) who successfully completed the SSC process. Data were available for all 18 officers, with no data lost.

Although there was a large standard deviation in both the successful and the unsuccessful groups for the officers’ anthropometric and performance measure data, those officers who were successful were significantly shorter (181.36 ± 5.35 cm, t (16) = 2.471, *p* = 0.025), lighter (85.36 ± 6.65 kg, t (16) = 3.118, *p* = 0.007), and performed better during the 2.4 km loaded march event (13.64 ± 0.92 min, *p* = 0.013) than the unsuccessful officers (Table 1). Conversely, the officers age, BMI, LMBI, UR, and 10 km loaded march results were not significantly different between groups (Table 1). Given the significant differences in performance between the two groups, it was not surprising that in the point-biserial correlations, being shorter (r_pb_ (16) = −0.526, *p* < 0.05), lighter (r_pb_ (16) = −0.615, *p* < 0.01), and faster (r_pb_ (16) = −0.572, *p* < 0.05) were correlated with course success.

## 4. Discussion

The aim of this study was to determine whether performance on a loaded explosive occupational task (the UR), a 2.4 km loaded movement, and a 10 km load carriage march were indicative of officer success on a five-day police SSC. The results of this study demonstrate that there was a significant difference between successful and unsuccessful officers in the SSC process, with officers of a shorter stature, lighter body weight, and presenting with a faster 2.4 km loaded march performance more likely to be successful. In contrast, there were no significant differences in the success of officers based on their BMI, LBMI, or performance in the anaerobic (UR) or longer load carriage march (10 km) events.

The findings reported in this study, whereby successful officers were shorter and lighter than their counterparts, are in contrast to those of Orr et al. [13]. In their study, Orr et al. [13] found that police officers who were successful at completing a SSC were taller and heavier than those who were not selected. However, when comparing the data, those who were successful in the study by Orr et al. [13] had a mean height of 183.80 ± 4.59 cm and weight of 91.40 ± 5.46 kg and those who failed were significantly shorter and lighter, whereas the successful officers in this study had a mean height of 181.36 ± 5.35 cm and weight of 85.36 ± 6.65 kg. This demonstrates that overall, those who were successful in both courses may generally possess similar anthropometric measurements, notably in height.

In this program of research, the UR and 10 km loaded march were not found to be associated with course success, while the opposite was true for the 2.4 km loaded movement. While limited research on anaerobic performance in the specialist tactical response police population makes it difficult to compare findings between studies, a study by Carlson and Jaenen [1] provides support for the findings of this study. Their study focused on Canadian special operations personnel [1], with a wingate anaerobic test being completed to calculate peak power output. The results of their study found that the results were not significantly correlated with successful selection. Acknowledging that no significant differences were found in this study in relation to the UR task and officer success, previous research has shown that explosive performance and mobility are negatively impacted by tactical load, in both general [36] and specialist [24] populations. On this basis, further research in this area in relation to selection of specialist personnel may be of value.

The results of this study for the 2.4 km loaded movement were comparable with those found by Hunt et al. [12], Pleban et al. [37], Zazanis et al. [38], and Teplitzky et al. [39]. For example, in the study completed by Hunt et al. participants were required to complete a 3.2 km battle run event while carrying webbing (7 kg) and a weapon within 16 min [12]. Additionally, a 5 km pack march was to be completed as fast as possible, without running or shuffling, while carrying webbing (40 kg) [12]. In both loaded events, performance in those who were successfully selected was significantly faster when compared to those who were unsuccessful. This potential association between run time and success has likewise been found when the participants were unloaded. In the studies by Pleban et al. [37], Zazanis et al. [38], and Teplitzky et al. [39], an unloaded 2 mile run (3.2 km) was completed as a component of the army physical fitness test (APFT) and was found to be either positively associated with success [37,38] or was a significant predictor of success [39] in U.S. Special Forces selection. A potential reason for both loaded and unloaded events to be predictive of success can be found in the works by Robinson et al. [30], who found aerobic fitness (as measured by a 20 m shuttle run) to be strongly correlated with load carriage times (5 km with a load of 25 kg), even up to 18 months later.

As is the case for anaerobic performance, there is a lack of research looking at performance in longer pack marches (>5 km) in the specialist population that could be compared to the results of the current study. Hunt et al. [12] reported the use of a 20 km loaded march (28 kg webbing while carrying a weapon) with a required completion time of 3 hours and 15 min. It was found that those who were successfully selected could complete the loaded march faster than the allocated time compared to those who failed. For general comparison, and given similar loads between the study by Hunt et al. [12] and this study, if the resultant times of the 20 km event of Hunt et al. [12] were halved to represent a 10 km loaded march, they are loosely comparable in nature to the results of this study. The mean times of completion for those who passed and failed in the study by Hunt et al. [12] were 91.45 and 96 min, respectively, while the mean time of completion for those who passed and failed in this study was 86.03 and 87.86 min, respectively. While both times in this study were faster than those of Hunt et al. [12], it is interesting to see the degree of separation between the successful and unsuccessful candidates, which may be more pronounced in longer events. As such, there is the potential for longer events to further widen the performance gap between groups. However, it must be noted that these times from Hunt et al. [12] are only broad estimates, and fatigue, the load carried, and the differences in terrain need to be taken into consideration.

Considering the potential for longer events, four studies were found to report increases in the energy costs (per unit of time) of carrying a load as task duration increased [40,41,42]. While these results were not always consistent, it is important to note that all four of these studies used a constant speed. A study by Patterson et al. [31] found the potential for energy expenditure could increase per unit of time by as much as 10 to 15% over durations of around 120 to 180 min while carrying a load of 27.5 kg. Studies by Patton et al. [43], Epstein, et al. [42], and Blacker et al. [40] reported significant (*p* < 0.005, *p* < 0.01, and *p* < 0.01 respectively) increases in energy cost per unit of time over long duration events (12 km and 120 min for the latter two studies, respectively) while their participants carried substantial loads (49.4 kg, 40 kg, and 25 kg respectively). However, of note is that neither Patton et al. [43] or Epstein et al. [42] found an increase in energy costs per unit of time over events of the same durations when participants carried lighter loads. This again supports the suggestion that longer distance loaded marches with heavier loads may be beneficial in the SCC process to increase the performance gap between SSC applicants.

One potential reason for the lack of findings for the UR and longer 10 km event may be the fitness levels of the officers. A critical review by Maupin et al. [44] found that elite tactical populations (military special forces and specialist police) generally possessed higher aerobic capacity scores when compared to the general population, police, or military. This is of note given that aerobic capacity was found to have stronger associations with load carriage performance than those of absolute and relative strength and power [30]. As such, the aerobic fitness of these officers illustrate that a ceiling effect could exist, whereby further improvements in aerobic fitness may no longer mitigate injury risk or ensure successful completion of the SSC. Thus, there would be other factors (such as those that are psychological in nature) that influence their success in the SSC and their subsequent injury rate [44].

This potential for a ceiling effect, as discussed here, has also been noted in studies by Orr et al. [13] and Hunt et al. [12] in specialist police and special forces populations, respectively. The results of the study by Orr et al. [13] found that height, body weight, pull-up scores, push-up scores, seven stage sit-up scores, and lift and carry speed were significantly and positively correlated with success in the selection process. However, it was found that aerobic fitness, as measured by a multi-stage fitness test, was not significantly associated with selection success. Hunt et al. [12] found that those who were successful in being selected were able to complete a greater number of push-ups and finish both the 3.2 km battle run and 20 km march in a shorter duration compared to those who failed. Conversely, it was again found that aerobic fitness, as estimated by the 20 m shuttle run test, was not indicative of success, as successful applicants had a mean VO_2_ of 55.1 ± 3.3 mL/kg/min, whereas unsuccessful applicants had a mean VO_2_ of 54.2 ± 2.8 mL/kg/min [12]. These findings provide sufficient evidence to support this hypothesis of a ceiling effect in aerobic performance as it relates to successful SSC selection. Furthermore, when the results of this study are compared to those of general police (mean VO_2_ of 37.50 mL/min/kg to 44.90 mL/min/kg [5]), those who failed the SSC still possessed an aerobic capacity that was indeed higher than the general police population.

There are some limitations regarding this study that should be acknowledged. Apart from the aforementioned potential ceiling effect of some measures, the small sample size is a limitation. This limitation is common in research in this population due to the specialist nature of these personnel and the limited pool of applicants. For example, in the study by Orr et al. [13], 17 police officers attempted the SSC. Considering this, data from the entire selection course was used for this study, and therefore represents 100% of personnel attempting this selection process; however, the results presented in this study should be interpreted with caution and may only be representative of this particular course.

The results of this study suggest that officers planning to attempt SSC may benefit from increasing their loaded 2.4 km run times. While the two other performance measures were not significantly associated with SSC success in this study, this may be due to the high level of fitness these personnel already possessed. As such, officers attempting selection should ensure that they have (and maintain) high levels of explosive and aerobic fitness. Finally, while lower body weight was associated with success in this population, attempting to lose weight to improve SSC success should be considered with caution, given that the results conflict with other studies.

## 5. Conclusions

Officers who were successful in completing an SSC performed the short distance (2.4 km) loaded movement event significantly faster than those who were not successful. Anaerobic performance in an explosive load carriage task (UR) and the time taken to complete the 10 km loaded march were not significantly different between those who did and did not complete the course. Given the high levels of fitness in the officers attempting selection, a ceiling effect may exist, whereby greater increases in, or higher levels of, fitness may not relate to selection success. Finally, additional research may be valuable to determine the benefit of including anaerobic performance measures and longer load carriage marches in future SSCs.

## Figures and Tables

**Table 1:** Descriptive statistics for the whole cohort, as well as successful and unsuccessful applicants.

Anthropometric Data/ Performance Measure.	Cohort (n = 18) Mean ± SD	Successful Applicants (n = 11) Mean ± SD	Unsuccessful Applicants (n = 7) Mean ± SD
Age (years)	32.11 ± 5.04	30.64 ± 4.97	34.43 ± 4.54
Height (cm)	183.72 ± 5.79	181.36 ± 5.35	187.43 ± 4.58 *
Body Weight (kg)	89.44 ± 8.56	85.36 ± 6.65	95.86 ± 7.45 **
BMI (kg/m^2^)	26.45 ± 1.58	25.94 ± 1.49	27.27 ± 1.46
Loaded BMI (kg/m^2^)	33.60 ± 1.61	33.33 ± 1.75	34.02 ± 1.38
Urban Rush (mins)	1.86 ± 0.15	1.87 ± 0.16	1.86 ± 0.15
2.4 km Loaded Movement (min: s)	14.28 ± 1.45	13.64 ± 0.92	15.29 ± 1.60 *
10 km Loaded March (min: s)	86.74 ± 2.40	86.03 ± 2.26	87.86 ± 2.34

Note: SD = standard deviation; BMI = body mass index. Significantly different from successful applicants at * *p* < 0.05 and ** *p* < 0.01.
